# Sustainable animal production: exploring the benefits of sperm sexing technologies in addressing critical industry challenges

**DOI:** 10.3389/fvets.2023.1181659

**Published:** 2023-11-23

**Authors:** Joana Quelhas, Patrícia Pinto-Pinho, Graça Lopes, António Rocha, Rosário Pinto-Leite, Margarida Fardilha, Bruno Colaço

**Affiliations:** ^1^Laboratory of Signal Transduction, Institute of Biomedicine (iBiMED), Department of Medical Sciences, University of Aveiro, Aveiro, Portugal; ^2^Department of Veterinary Clinics, Institute of Biomedical Sciences Abel Salazar-University of Porto, Porto, Portugal; ^3^Department of Veterinary Sciences, University School Vasco da Gama, Coimbra, Portugal; ^4^Centre for the Research and Technology of Agro-Environmental and Biological Sciences (CITAB), University of Trás-os-Montes and Alto Douro, Vila Real, Portugal; ^5^Laboratory of Genetics and Andrology, Hospital Centre of Trás-os-Montes and Alto Douro, E.P.E., Vila Real, Portugal; ^6^Experimental Pathology and Therapeutics Group, Portuguese Oncology Institute of Porto FG, E.P.E. (IPO-Porto), Porto, Portugal; ^7^Department of Imuno-Physiology and Pharmacology, Institute of Biomedical Sciences Abel Salazar-University of Porto, Porto, Portugal; ^8^Animal and Veterinary Research Centre (CECAV)/Associate Laboratory for Animal and Veterinary Sciences (AL4AnimalS), University of Trás-os-Montes and Alto Douro, Vila Real, Portugal

**Keywords:** sperm sexing, reproductive technologies, reproduction, animal production, animal welfare, sustainability

## Abstract

The sex of the animals is of paramount importance in many animal production systems. This is particularly evident in the production of milk or in breeding programs focused on the production of female animals. In some cases, slaughter or euthanasia of animals of the unwanted sex becomes the only solution, highlighting ethical and economic concerns. As global demand for food continues to rise, the importance of addressing these issues becomes more evident. Reproductive technologies, such as sperm sexing techniques, may hold the key to addressing both animal welfare and the sustainability of animal production. The use of semen enriched with sperm capable of producing offspring of the desired sex can serve as a valuable tool for producers to exert greater control over production outcomes, not only helping to mitigate welfare issues related to the unnecessary premature death of unwanted offspring but also providing a possible ally in the face of stricter animal welfare guidelines. In addition, sexed semen can also contribute to financial gains and reduce greenhouse gas emissions and food waste associated with the less profitable part of the herd. This paper explores the positive impacts that sperm sexing can have on animal welfare, economy, and environment. It also discusses currently available options and strategies for more successful implementation of sexed semen. Partnerships between companies and scientists will be essential to find innovative ways to adapt current production systems and develop sperm sexing technologies that apply to most livestock industries.

## 1 Introduction

The global food market is growing, as well as the demand for sustainable and animal welfare-focused production systems. The animal production sector has been associated with numerous scenarios that might compromise animal welfare and is commonly considered a relevant source of greenhouse gas (GHG) emissions despite this being a controversial topic, with opinions and statistics varying greatly among authors (1–3). These problems challenge both the animal production sector and scientists in the search for innovative ways of adapting the current animal protein supply systems.

The use of reproductive technologies, such as artificial insemination (AI), has aided animal production for high efficiency (4). In productions with a preference for one sex over the other, the implementation of sexed semen would allow for pre-selection of the offspring sex and, consequently, redirect production and increase efficiency and profitability (5, 6). However, this technology is predominantly established in cattle. Sexed semen sorted by flow cytometry or gender ablation with an accuracy greater than 90% and with fertilization rates similar to those of unsexed semen is already available for sale (7, 8). For other species, the utilization of semen sorted by flow cytometry remains unviable or generally unprofitable (5). The quantity of spermatozoa per straw is lower compared to what is considered an ideal insemination dose and the sperm quality is negatively affected, which may impair fertility (9). Therefore, these disadvantages limit its applicability to a broader range of species. In certain cases, the use of sexed semen has only been successful when large inseminating doses were used, or, in the case of small doses, when AI was performed by laparoscopy, which requires a greater financial investment in equipment and specialized technicians (10, 11). Nevertheless, in the last decade, some solutions have begun to emerge in the market for species such as goats, sheep, pigs, horses, and dogs, and further research is warranted develop more affordable sexing technologies aiming at obtaining good quality semen doses (12, 13).

In this review, we will discuss the positive impacts that sperm sexing can have on animal welfare, economy, and environment, along with its limitations. We will also explore currently available sperm sexing techniques and strategies aimed at a broader implementation of AI with sexed semen.

## 2 Demand for protein of animal origin: what is the future?

**Figure 1:**
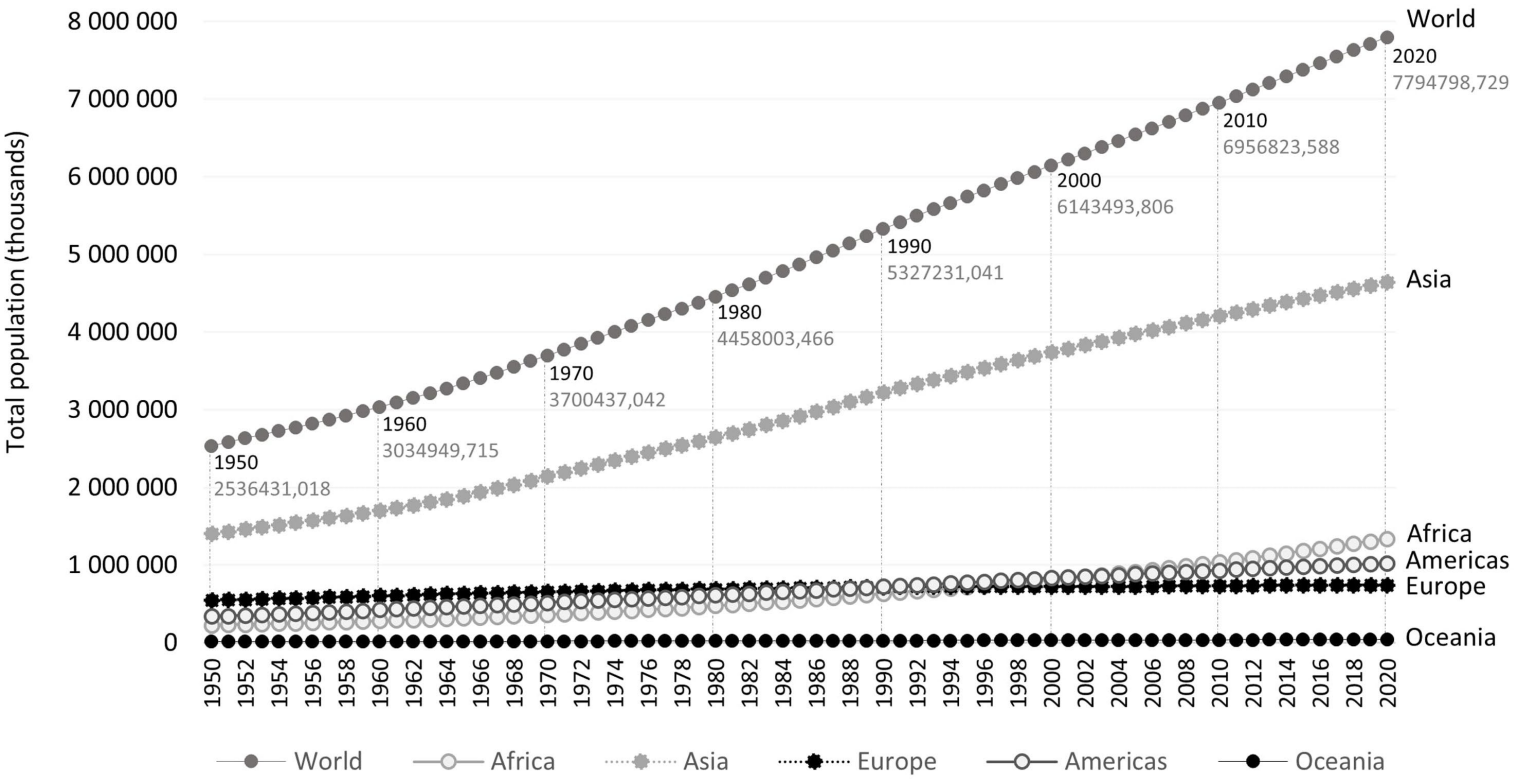
Total population estimates, between 1950 and 2020, in the world and by region (Africa, Americas, Asia, Europe, and Oceania), according to the United Nations database for World Population Prospects, accessed 2022-04-05 (14).

Although the world population growth rate peaked in 1962–1963 (2.2%) and has been declining since then, the world population is still growing rapidly (Figure 1) (15). A new significant population peak was reached in November 2022. There are currently about 8.0 billion people living on Earth and estimates are that there will be 10.4 billion by the 2080s (15, 16).

With the world population steadily growing and consumption rates on the rise, the necessity of ensuring a greater food supply, especially from protein sources, becomes increasingly evident. According to calculated data based on the latest estimation of the Food and Agriculture Organization of the United Nations (FAO) (Supplementary material S1), the average protein supply in the world increased by 10% (the equivalent of 7.5 g/capita/day) between 2000 and 2018 (17). In some regions like Africa or Asia, this increase was even more striking, reaching 13 and 15%, respectively (17).

**Figure 2:**
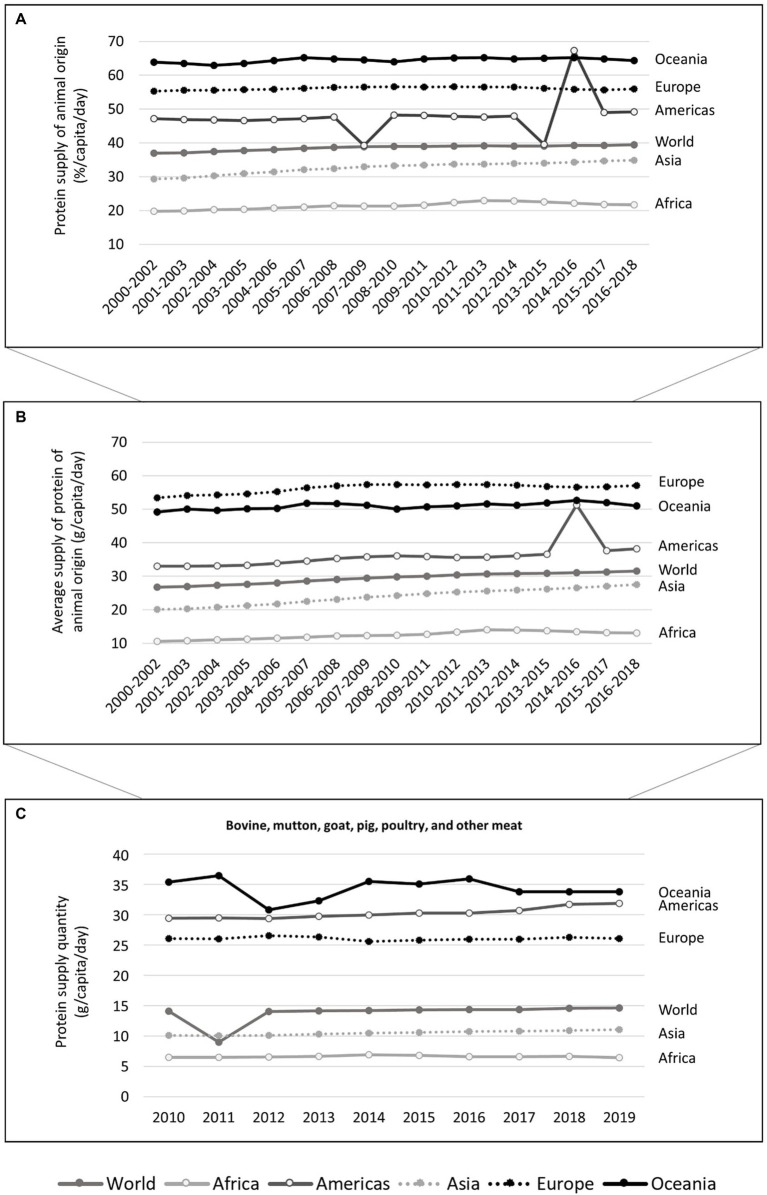
Percentage of the protein supply *per capita* per day that it is from animal origin **(A)** and the average protein supply of animal origin (measured in g *per capita* per day) **(B)** between 2000 and 2018 (3 years-average), and the protein supply quantity (measured in g *per capita* per day) **(C)** in terms of bovine, mutton, goat, pig, poultry, and other meat between 2010 and 2019, in the world and by region (Africa, Americas, Asia, Europe, and Oceania), according to the FAO database, accessed 2022-02-21 (17). It includes calculated data (Supplementary material S2).

Animal-source foods provide proteins containing all amino acids in adequate quantities for human consumption and a wide variety of minerals, improving human nutrition and health (18). For example, deficiencies in some micronutrients present in meat were already linked to brain-related disorders. Some vegetables contain iron, zinc, and omega-3 fatty acids, but at lower amounts compared to animal sources [reviewed in (19)]. This may be one of the reasons why protein of animal origin accounts for a sizable share of overall protein consumption. Animal-source foods have already been linked to better cognitive performance (20). Between 2000 and 2018, about 38.5 ± 0.8%/capita/day of the protein supply in the world was of animal origin, with regions like the Americas, Europe, and Oceania registering about 47.8 ± 5.6%, 56.1 ± 0.4%, and 64.5 ± 0.7%, respectively (calculated based on FAO data (17); Supplementary material S2). An overview of the supply of animal-derived protein from 2000 to 2018 is provided in Figure 2.

**Figure 3:**
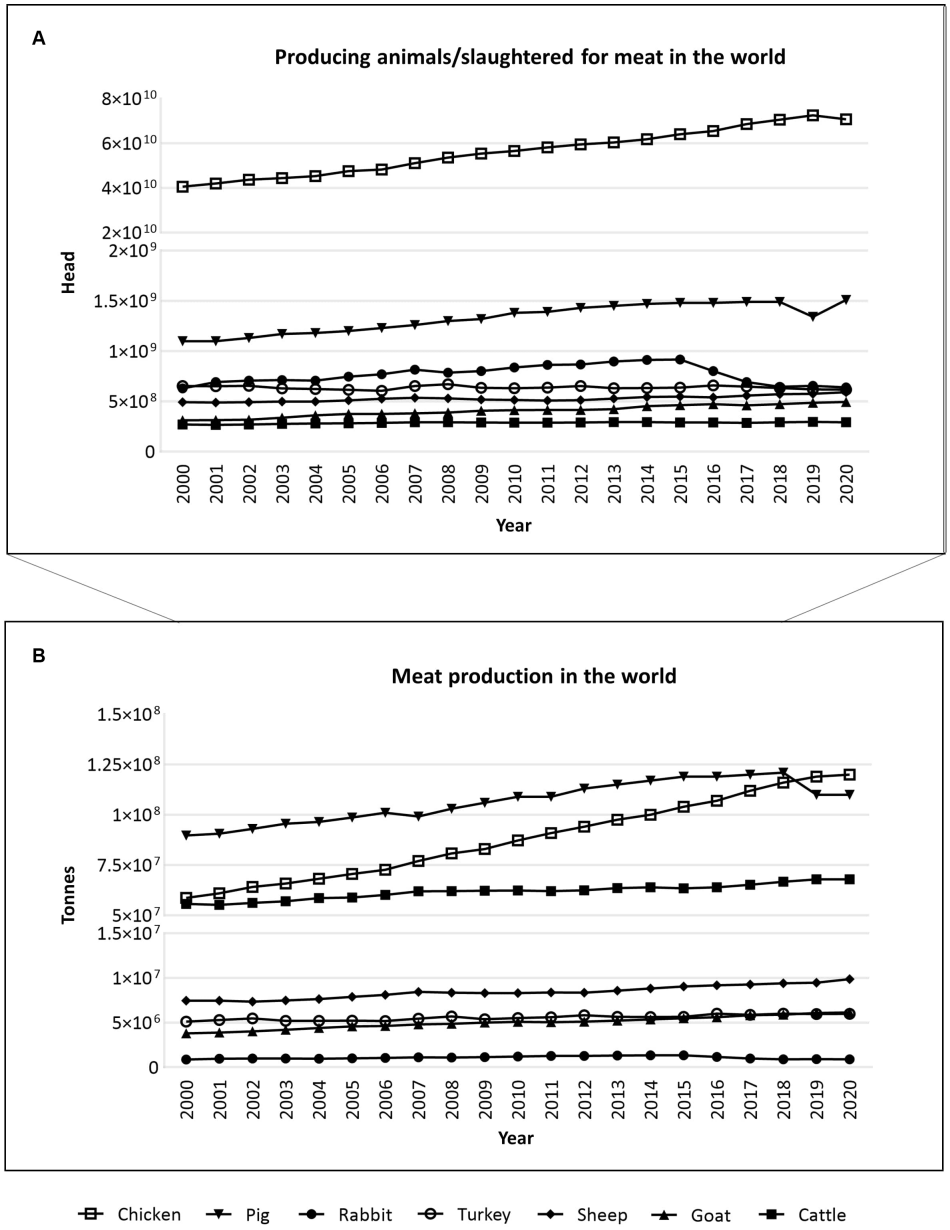
Animals produced/slaughtered (measured in heads) **(A)** and meat production (measured in tonnes) **(B)** for meat consumption in the whole world, between 2000 and 2020, according to the FAO database, accessed 2023-02-18 (22). According to FAO, it may include official, semi-official, and estimated, or calculated data. Raw data is available in Supplementary material S3.

Meat is still one of the most popular high-quality protein sources, alongside milk, eggs, and fish (21). Every year, millions of animals are slaughtered for meat around the world (Figure 3; Supplementary material S3). Among some of the main species for meat consumption, such as cattle, goats, pigs, sheep, chicken, and turkey, chickens stand out massively, with about 70.8 billion slaughtered for meat in 2020, according to FAO (Figure 3A) (22). Pigs come next (~1.5 billion), followed by rabbits (~639.8 million) (Figure 3A). When it comes to meat consumption in tonnes, between 2000 and 2020, pigs and chickens once again stood out, followed by cattle (Figure 3B) (22).

Meat consumption in most European nations is currently ranging between 70 and 90 kg *per capita*. Some countries consume more than 100 Kg of meat *per capita*, such as Australia or the United States of America (USA) which has a considerable *per capita* intake of 120 Kg (23). Meat consumption by country appears to be positively associated with their economic growth throughout time. In only 22 years, the global consumption of beef and veal, pig, poultry, and sheep per year climbed by 107 million tonnes in the World, growing from 227 million tonnes in 2000 to 334 million tonnes in 2022 (24). Overall, the trend is to increase in the coming years (21). According to FAO estimates, global meat consumption is expected to reach an average of 455 million tonnes by 2050 (25). However, several concerns arise with it.

Animal welfare, pollution, land and water use, and inherent biodiversity loss are just a few of the aspects that our current governments and society are concerned about, when it comes to intensive livestock production (1). When it comes to GHG emissions, it can be challenging to determine the exact levels attributed to livestock, and, therefore, the numbers remain the subject of ongoing debate and controversy in the scientific literature. The discrepancy in values can be attributed to various factors, including geographic location, the scope and subsections considered as part of livestock emissions, and the models used for predictions, with studies that account for all aspects of livestock production at a global level and others that are more centered in national emission statistics (26). For that reason, there is a wide range of values reported in the literature, with some authors pointing to higher emissions associated with the livestock production sector (27, 28), while others suggest that livestock contributes less than 3% of total anthropogenic GHG in the USA (26).

Apart from CO_2_, methane (CH_4_), and nitrous oxide (N_2_O) are reported as the most relevant GHG from animal agriculture, with an estimated impact on global warming 28 and 265 times higher than CO_2_, respectively [reviewed in (2)]. Also, animals grown for meat production require feeding. Around 40% of the wheat and corn produced in the world is utilized for this purpose (23). Given the rise in meat consumption, it is anticipated that at least 1.45 million tonnes of cereals will be employed as animal feed in 2050, representing the yearly calorie need (1 million Kcal/person/year) for more than 3.5 billion people (29). If these projections prove true, we will need in 2050 1.52 to 1.59 times the amount of wheat and corn currently produced (23).

In fact, we should not have to choose between producing enough food for the whole population and protecting our planet’s future (30). This is what defends the recent European Union Farm to Fork strategy, which aims “to make food systems fair, healthy and environmentally-friendly” (30). Among others, there are discussion of how the European Union’s promotion program may support carbon-efficient animal production systems and how to drive a transition to healthier diets while reducing meat consumption. Other strategies have been advocated, such as organic meat production, or more extreme approaches such as prioritizing vegetarianism and meat substitution with synthetic meat and meat analogs (31).

Nonetheless, the decision to restrict meat intake should be made with caution. This might not be the greatest global solution. Animal-source foods are lacking in the diets of almost 800 million people and are critical not only to improving health but also to the world economy (1, 32). Therefore, it is in the interest of the animal production industry to discover new solutions to ensure that consuming meat remains a healthy, eco/animal-friendly, and viable option in the future. Reproductive technologies might be the key.

## 3 Animal production systems and the role of reproductive technologies

Currently, animal management and reproductive improvement are essential to face competitiveness between companies and ensure economic gains. In fact, animal production is almost inextricably linked to biotechnological and assisted reproductive technologies (ARTs) (4). AI and *in vitro* embryo production are common examples (33, 34). Another option is sex pre-selection, which is based on the use of sexed semen for insemination (widely used in cattle production) or on the pre-selection of the sex of *in vitro*-produced embryos prior to transfer (33).

These techniques reduce generation gaps, increase access to genetics from top-performing animals, minimize the spreading of infectious or contagious illnesses between animals, and enable the protection of endangered animal species (35). However, their application varies between species. Cattle, pigs, sheep, goats, and rabbits may be the species for which AI is most accepted. Among others, AI is also used on dogs and horses, but it attracts less attention [reviewed in (36, 37)]. In the horse industry, despite AI being used in sport horses to breed top performers, some breed associations only accept registering the horse if it was conceived by natural breeding [reviewed in (36)].

Despite all the mentioned advantages, animal welfare and the company’s earnings may yet be improved by using new or improved reproductive technologies related to sex pre-selection. In some cases, there is a preference for one sex over the other due to differences in meat quality or ability to produce milk, or simply for the fact that some productions are directed towards either males or females (5, 38–41). Therefore, sexing technologies could significantly benefit animal production systems, particularly in the case of cattle, swine, rabbits, and poultry, given the high annual slaughter rates of these species for meat consumption (Figure 3).

### 3.1 Sperm sexing: an innovative tool for more sustainable systems

The perfect scenario would be to have an efficient, simple, and inexpensive approach that would allow the dissemination of sustainable productions from an animal welfare point of view, while also ensuring economic returns. Among the several ARTs listed, sperm sexing combined with AI has great potential to meet all these requirements. Sperm sexing *per se* allows the separation of a semen sample into two fractions, one carrying a large majority of X chromosome-bearing spermatozoa (X-sperm) and the other holding a large majority of Y chromosome-bearing spermatozoa (Y-sperm) (42). As a result, by employing sexed semen samples in AI programs, it is feasible to pre-select the offspring sex. Implementing this approach could lead to streamlined herd management, potentially accelerated herd expansion and genetic progress, and maximized use of resources, all while reducing wastage, as it will be further discussed (6, 42, 43). Therefore, it might be valuable to apply it to species other than cattle (5, 33, 41).

The state of the art regarding sperm sexing attempts and technologies is widely reviewed (41, 44–46).

Currently, sperm sorting by flow cytometry is the most accepted methodology. Semen is sexed based on the difference in DNA content between the sex chromosomes since the X chromosome has more DNA content than the Y chromosome. For example, in cattle, the X-sperm has about 3.9% more DNA than the Y-sperm, varying slightly among breeds (47). The technique was patented in 1992, improved, and employed in the US and Europe, but only for beef and dairy breeds (48, 49). Semen straws with 2 million sexed spermatozoa are widely commercialized with over 85–95% accuracy, and straws with 4 million are also available under the trade name SexedULTRA 4 M (49, 50). As a result of the development of extenders and the implementation of SexedULTRA^™^ procedures, caprine sexed-sorted semen has also been commercially available since 2015, with a female sex purity of approximately 93% (51). Similarly, in 2018, ovine sexed-sorted semen doses became available, usually ranging from 2–6 million spermatozoa with a female sex purity exceeding 90% (51, 52). However, this procedure requires expensive equipment and specialized technicians, and it is still considered time-consuming (50). The process also negatively impacts semen quality, mostly because the external labeling combined with the exposure to laser light can damage or even kill the sperm cells (53, 54). Consequently, the conception rate and the number of fetuses tend to be negatively affected, especially if no further quality selection process is carried out (51, 52, 55).

Several attempts have been made to find other techniques for sexing semen, like albumin gradients, Percoll gradient, swim-up, and H-Y antigen, but they have several limitations (42). De Luca et al. suggested Raman spectroscopy for sperm sexing based on biochemical differences mainly at the nucleus level (53). Centrifugation or microfluidics coupled with UV-absorbance spectroscopy may be another solution, based on possible X- and Y-sperm differences regarding density, charge, and shape (56, 57). High-precision techniques used for nanoparticle separation, capable of picking up nanosized differences, such as flow field-flow fractionation (FIFFF), are also promising options (58). So far, they are not yet implemented and/ or are still expensive.

Some other technologies have shown up in the market recently. In 2017, ABS Global company launched Sexcel sexed genetics^™^, a gender ablation technology marketed for X-sorted semen of Holstein, Jersey, Norwegian Red, Angus, Red Angus, Brangus, Hereford, Simmental, Gyr, and Nelore cattle breeds (7, 59). It stains DNA with Hoechst 33342 to differentiate the X- and Y-sperm, and a laser destroys the Y-sperm (accuracy of around 85%) (36, 43, 60). Contrarily to flow cytometry, it does not divide cells into droplets and does not require cell steering, hence an electric field is not required (43). Additionally, the remaining cell debris does not appear to affect the conception rate (61). In 2020, gender-ablated semen performed at 78% of unsexed semen in a field trial with beef heifers and cows. We should take into account that the gender-ablated semen straws had 1.25 million normal progressive sperm post-thaw and the unsexed semen straws had approximately 9.17 million (60). The company advertises even better results, stating that compared to unsexed semen, Sexcel^™^ achieves a 90% relative conception rate to conventional semen (62). They also refer relative conception rates 3% (heifers) to 3.3% (cows) higher than those obtained with other sexed semen, based on more than 840.000 (>600 farms) and 280.000 (>450 farms) inseminations, respectively (43).

Another novelty is the sperm sexing kits from Nuri Science Inc. (12). These kits are based on a patented additive containing an antibody that binds to a protein on the cell membrane of the head of Y-sperm, promoting their agglutination (63). Since Y-sperm agglutinates, X-sperm can fertilize more rapidly, thereby increasing the probability of a female pregnancy. Nuri Science Inc. offers commercially available WholeMom kits for bovine, equine, caprine, ovine, canine, and porcine species. The accuracy of these kits for predicting female gestations varies from 70% (in sows) to 90% (in heifers), depending on the species. They also offer a WholeMan kit designed to promote male gestations with an accuracy of over 90%, specifically for use in bulls, where the addition of a specific additive enhances the motility of Y-sperm over that of X-sperm. The company describes simple procedures, such as mixing the semen sample with the commercial vial and carrying out AI, in the case of the WholeMan kit, or mixing, incubating for 20–30 min, and then conducting AI, if using the WholeMom kit intended for heifers. Some variations are employed, such as an overnight incubation when using the WholeMom kit for sows (12, 64).

Aligning with this option, EMLAB Genetics kits have emerged, providing the flexibility to sex both genders and allowing sperm sexing of fresh and frozen-thawed samples for the same species as the WholeMom kits (13). In this case, the company offers several additives that can enhance the fertility and motility of X-sperm while ‘slowing down’ the Y-sperm, and vice versa. However, not much information is shared regarding the scientific basis of this process. Therefore, when the enriched sperm doses are used, spermatozoa are ‘sorted’ in the reproductive tract and more ova are fertilized by spermatozoa of the sex of interest. Regardless of the species, it is only necessary to incubate the semen sample with the additive for 10 to 30 min, at 32–38.6°C, depending on the protocol, being then ready for insemination (13). According to EMLAB’s data, sperm sexing accuracy for female gestations for bovine is 75–90% if using fresh semen and 70–75% if using post-thaw semen; for equine is 65–85% if using fresh/cooled semen and 75% or 90% (depending on the product) if using post-thaw semen; for caprine is 75–90% if using fresh semen and 80–85% if using post-thaw semen; for ovine is 75–90% for both fresh and post-thaw semen; for canine is 75–85% either using fresh, cooled, or post-thaw semen; and for porcine is 80–90% for both fresh semen and post-thaw semen (13). Regarding the average sperm sexing accuracy for male gestations, for bovine is 75–90% if using fresh semen and 75% if using post-thaw semen; for equine is 65–85% either using fresh, cooled, or post-thaw semen; for caprine and ovine is 75% if using post-thaw semen; for canine is 75–85% either using fresh, cooled, or post-thaw semen; and for porcine is 80–90% for both fresh semen and post-thaw semen (13). The company also claims fertility is boosted by 5–25% when using their product, depending on the species (13).

Sexing technologies are also of interest to poultry producers since there are sex-related issues in egg production (65). Male layer chicks do not produce eggs and are not suitable for meat production due to their slow growth compared to broilers (65, 66). Therefore, culling at 1 day old by maceration or gasification and use of the carcass for animal feed production is also the unique outcome for the majority (67). Around 330 million day-old male chicks are being killed in hatcheries every year solely in the European Union (67). However, in contrast to mammals, male birds are homogametic, meaning that they possess two Z chromosomes, while females are heterogametic, with Z and W chromosomes [reviewed in (38)]. Therefore, since the sex of the offspring is determined by the female, sperm sexing technologies are not currently an option. Highly sensitive in-ovo sexing technologies are being considered, such as the use of visible near-infrared point spectroscopy and two-wavelength fluorescence spectroscopy, or even the control of the environmental conditions of incubators to induce male-to-female conversion (68–70). Other techniques are reviewed by Gautron et al. (38).

While several sperm sexing options for various species have been discussed, the practical implementation of these techniques on a large scale in most farms may be limited by certain constraints. For example, the first practical limitation may be related to the few commercially available options and their respective accuracy in sorting X- and Y-sperm (summarized in Table 1), which implementation may or may not compensate depending on the profit potential associated with each animal. Rabbits currently lack commercially available options, and for species such as ovine, the availability of sex-sorted semen is limited due to a shortage of commercial sorting facilities worldwide (51, 52). Between 2018 and 2020, laboratories were established in the USA, United Kingdom/Europe, South Africa, and Australia/New Zealand for ram sperm sexing. Nevertheless, it is necessary to transport rams to the sorting facility for semen collection, and the AI procedure should take place at a location where the collected semen can be delivered within about 12 h after processing (52).

**Table 1:** Commercialized sperm sexing options for various species and their accuracy.

Technique	Technique principle	Target specie	Cellular type	Accuracy (%)
Flow cytometry	X-sperm has more DNA content than Y-sperm, allowing them to be sorted	Bovine	X-sperm	75% and 85–95%
			Y-sperm	75% and 85–95%
		Caprine	X-sperm	93%
		Ovine		>90%
Sexcel sexed genetics^™^	Gender ablation	Bovine	X-sperm	85%
WholeMom kit (Nuri Science Inc.)	An antibody promotes the agglutination of the Y-sperm, slowing them down	Bovine	X-sperm	90% (heifers)
		Equine		Not specified
		Caprine		Not specified
		Ovine		Not specified
		Canine		Not specified
		Porcine		70% (sows)
WholeMan kit (Nuri Science Inc.)	An additive enhances the motility of Y-sperm over that of X-sperm	Bovine	Y-sperm	90%
EMLAB Genetics’ kits	An additive enhances the fertility and motility of X-sperm while slowing down the Y-sperm	Bovine	X-sperm	75–90% (fresh semen) 70–75% (post-thaw semen)
		Equine		65–85% (fresh and cooled semen) 70–75% (post-thaw semen)
		Caprine		75–90% (fresh semen) 80–85% (post-thaw semen)
		Ovine		75–90% (fresh or post-thaw semen)
		Canine		75–85% (fresh, cooled, or post-thaw semen)
		Porcine		80–90% (fresh or post-thaw semen)	An additive enhances the fertility and motility of Y-sperm while slowing down the X-sperm
		Bovine	Y-sperm	75–90% (fresh semen) 75% (post-thaw semen)
		Equine		65–85% (fresh, cooled, or post-thaw semen)
		Caprine		75% (post-thaw semen)
		Ovine		75% (post-thaw semen)
		Canine		75–80% (fresh, cooled, or post-thaw semen)
		Porcine		80–90% (fresh or post-thaw semen)

Also, the success of fertilization *per se* using sexed semen can be influenced by several factors. Although sexed insemination doses contain significantly fewer spermatozoa compared to unsexed semen doses, the success of the insemination may be primarily determined by the quality and total number of viable spermatozoa per insemination dose (51, 71, 72). The timing of insemination should also be considered since sex-sorted spermatozoa have a shorter lifespan than unsexed semen and complete the capacitation process sooner due to the semen-sorting process that partially induces capacitation-like changes [reviewed in (8)]. Other factors, such as the age and reproductive history of the animal, the type of ART used, synchronization protocols, estrus presence or absence, preovulatory follicle size (POFs) in the case of cattle, hormone-like substance administration, and even the technicians and heat stress may further impact fertility and pregnancy rates (9, 60, 73–77). These factors may also underlie the variations in success observed across studies and species when employing sexed semen for animal reproduction.

In summary, despite recent advancements, research on new or improved sperm sexing methods and other reproductive technologies must be a priority in the field in the upcoming years, moved by the potential of this technology to improve animal production.

## 4 Implications of sperm sexing technologies in animal welfare, economy, and sustainability

The widespread implementation of sperm sexing technologies in the animal production sector would contribute to meeting goal number 9 (build resilient infrastructure, promote including and sustainable industrialization, and foster innovation) and goal number 12 (ensure sustainable consumption and production patterns) of the United Nations’ 2030 Agenda for Sustainable Development (78). The potential for a significant and favorable impact of sperm sexing on animal welfare, on the economy of the companies, and even on the environment will be explored further below.

### 4.1 Animal welfare

It is undeniable that animal welfare is an important aspect of animal production, and its definition does not differ greatly across the population, at least in Europe. According to a survey carried out in 2015 among 27,672 citizens of 28 members of the European Union, 46% of the participants defined animal welfare as the need to respect all animals and 40% as the way farmed animals are treated in an attempt to provide them with a better life (79). Based on the same survey, evidence suggests that the animal production sector should take animal welfare more seriously in their management policies. A significant majority of 94% of respondents expressed the belief that safeguarding the welfare of farmed animals is essential. Additionally, 82% of the participants agreed that animal welfare should be better protected than it is in the present state and 62% strongly agree that imported products from outside the European Union should adhere to the same animal welfare standards as those within the European Union (79).

In fact, for many years, the well-being of farm animals was essentially perceived and evaluated in terms of their productive potential (80). However, despite awareness and technological improvements, numerous problems still constantly disturb the guarantees of welfare, such as housing conditions, separation of mothers and offspring, stress related to transport, and social isolation (80–82). A recent brief communication points out welfare issues *per se* in this industry after observing that, among 355 male dairy calves, 20% arrived at auctions and calf-rearing facilities in North America with at least one health problem (83). According to the authors, calves in suboptimal conditions can be sold, for example, for 23 to 90% less than predicted depending on the breed and genetics, or even not sold at all (83).

Animal welfare can be threatened in other ways and literature reports several weaknesses where the use of sexed semen may benefit animal well-being. One of them is the birth of animals of less productive sex, such as male calves, goats, and buffaloes on dairy farms (40, 66). Nonetheless, the rearing costs coupled with the low or no economic value of the animals have already made euthanasia after birth an occasional outcome for male dairy calves (84–86). Several initiatives are now ongoing with the ambition of ending the euthanasia of calves by 2023 and promoting responsible breeding strategies to minimize the number of calves born without a market (85). Partnerships between dairy and beef productions are one of those initiatives, allowing the use of surplus male dairy calves for meat. Coupling this with the use of sexed semen is also a promising mitigation strategy (86). Also in the dairy goat industry, buck kids are either sent to fattening facilities, where they mix them with kids from various farms, increasing stress and disease risks, or they are fattened on the farm, which may result in suboptimal conditions for them due to space constraints. Proper care practices, such as colostrum provision and hygiene, are vital but may be overlooked because buck kids are not a significant source of income for farmers. The same may happen for surplus males of other species, impacting their welfare and health [reviewed in (87)].

In swine production, sperm sexing technologies can also be beneficial. Castration has traditionally been a common solution to address the aggressive sexual behavior of male pigs and the development of a pungent odor in their meat, known as boar taint (88). Nevertheless, castration is often performed without pain treatment or anesthesia, which poses associated risks and discomfort for the animals. This practice is currently under increased scrutiny (89). Even if it depends on each producer’s goals, by using sperm sexing technologies, producers would have the option to prioritize the production of female pigs, thereby minimizing both situations and contributing to improved welfare conditions in production systems (90).

The growing global demand for food also puts pressure on livestock to intensify production, which, in turn, threatens animal welfare (82, 83). In cuniculture, it is common to employsemi-intensive systems lasting 42 days, where does are inseminated or mated 11 days after parturition. However, certain studies have shown that extensive systems lasting 56 days (with a 25 days pause) offer advantages, such as better body energy balance (91), improved overall body condition (91, 92), increased fertility rates (93, 94), and reduced kitten mortality (95). Some also argue that this type of system, which involves the insemination of does only after weaning, may be more suitable for primiparous does than intensive systems (93). Nevertheless, if there is a preference for one of the sexes, the use of sexed semen of comparable quality to unsexed semen would be a valuable tool, allowing for obtaining the usual number of desired animals in a shorter timeframe. This would be possible since every gestation would result in the birth of a large majority of animals of the intended sex, contrary to what happens when unsexed semen is used, where the odds are 50–50% (5). Furthermore, in polytocous species like rabbits, even sexed semen doses with lower accuracy rates can be beneficial, as a lower but consistent deviation will still significantly increase the production of the desired sex (5). Therefore, the introduction of sexed semen of adequate quality in cuniculture might have the potential to reduce the number of females required for breeding per farm, while still achieving the desired number of animals of the intended sex per year..

Also, both heifers and cows commonly experience constraints such as dystocia, which refers to abnormally protracted or slow labor (96). Using sexed semen may provide a potential solution to mitigate this issue (97). Female calves are smaller than male calves. Hence, these incidents could be reduced with the use of X-sperm (98). According to some reports, the utilization of sexed semen reduced challenging births by 28% for heifers and 64% for cows, as compared to unsexed semen (97).

### 4.2 Economy

Despite the use of sexed semen being more expensive than unsexed semen, in many cases it tends to be economically justified (42, 99). One of the major advantages lies in the potential utilization of such technology in breeding farms that specialize in selling replacement females or males, or in markets that may have a preference for one gender, such as dairy production and breeders of certain horse and dog breeds (5, 41, 56, 76, 90). For those, the ability to choose the sex of the animal would be a tremendous asset in directing production exclusively towards the desired offspring, thereby improving efficiency and providing a competitive advantage and potentially higher profits for producers (5, 41). In dairy farms, female dairy calves are more valuable than male dairy calves. As an unwanted by-product of breeding with conventional semen, the surplus male animals can be exported for veal production or reared for meat production, but producers have low monetary returns with them (6, 86). Furthermore, in cases where producers opt to euthanize male dairy calves, their carcasses are usually simply destroyed (66). Events like this represent relevant economic losses for producers, considering the production and feeding costs they have with the mothers. In the case of rabbits, females intended for reproduction are also significantly more valuable than the males born in their respective litters. Additionally, these males lack the ideal characteristics for meat production, resulting in little to no profit for producers (5, 41). When it comes to horse breeding, the choice of gender preference may vary by discipline. In the context of Polo sports, Quarter horse racing, and cutting horses, mares are typically preferred over stallions, whereas for Thoroughbred racing, dressage, reining horses, and show jumping, males are generally preferred over mares. Consequently, the same benefits apply in these cases [reviewed in (76)]. Some dog breeders also have a preference for one of the sexes, and, as such, combining sperm sexing with their polytocous nature, would offer the potential for market improvement through the production of larger same-sex litters (56). Nonetheless, it is noteworthy that research exploring sexed semen application in dogs remains particularly scarce (56, 100). Therefore, overall, the possibility to pre-select the preferred sex has the potential to improve the efficiency of these farming systems (42, 97). Other critical aspects that highlight how farm economies can benefit from this reproductive technology will be explored, as well as the challenges encountered.

#### 4.2.1 Cattle

Economic benefits of using sexed semen in dairy productions also include the added value of cross-bred calves not used as substitutes, enhanced herd turnover rates leading to cost savings, reduced expenses associated with dystocia management, and increased genetic progress rate (101–103). The possibility of selecting the sex of the animals may be, therefore, decisive for farm profitability in beef and dairy cattle (42, 104) and may help to mitigate the challenges that we have already addressed (6, 83).

According to our experience, the cost of bovine sexed semen above unsexed semen can be easily about 20€ per dose (Table 2), which discourages many producers from implementing it. A recent pilot survey also demonstrated that farmers were not up to date on the advantages and current advances in sperm sexing technologies (86).

**Table 2:** The average price ranges for bovine unsexed and sexed semen doses with different sperm number and accuracy, according to our experience and business partners.

Production purpose	Average price ranges per dose of semen				
	Unsexed (8 M)	Sexed X (2 M; A.75%)	Sexed X (2 M; A.90%)	Sexed X (4 M; A.90%)	Sexed Y (2 M; A.90%)
Milk	9–30€	12–14€	23–42€	41–55€	NA*
Meat**	5–6€	NA*	NA*	NA*	26–36€

Semen straws can have 2 million (2 M), 4 million (4 M), or 8 million (8 M) spermatozoa, and be sorted with an average accuracy of 75% (A.75%) or 90% (A.90%). *NA, not available and **Common breeds: Aberdeen-Angus and Limousine.

Several studies, primarily developed in cattle, have compared the tangible economic benefits of using ARTs, such as AI, with sexed and unsexed semen, emphasizing the advantages of preselecting the sex of offspring (99, 102, 105). A model-based study in 2007 calculated the expected net present value of AI with (un)sexed semen in dairy heifers (106). They considered three different scenarios, where the conception rates when using sexed semen would be 53, 75, and 90% of those achieved when using unsexed semen (106). They concluded that the net present value of sexed semen would only compensate when conception rates were 90% of those achieved with unsexed semen (106). Conception rates of this order for sexed semen are rather optimistic, although already described in an unpublished field trial that compared fresh sexed semen with frozen unsexed semen (Livestock Improvement Corporation (LIC) 2012, as cited by Butler (107)). However, other possible biosecurity and animal welfare-related benefits of its use that were not taken into account in those models must be weighed when evaluating the pros and cons of sexed semen implementation (106). Furthermore, according to Osada and colleagues, the utilization of sexed semen in a dairy farm holds the potential for significant benefits. It has been shown to improve the birth rate of heifers, thus enabling a more refined selection process for replacement females based on their genetic potential for milk production (102). Also, in their study, using sexed semen provided a more profitable price per female calf than using unsexed semen, as the higher female production rate offset the decreased conception rates. Nevertheless, according to the authors, special attention should be paid to maintaining the standards of cattle rearing when using frozen-thawed X-bearing semen, so that the pregnancy rate does not decrease (102). A previous study on Holstein heifers also revealed better results when using sexed semen compared to unsexed semen (105). Although the conception rate was slightly lower when sexed semen was used (31.6% vs. 39.6%), it allowed a yield of 86% heifers instead of only 48% obtained for unsexed semen. Abortion rates (6.1% vs. 6.5%) were similar (105). Still, this was a retrospective study where the same conditions were not always used and, therefore, the impact on the conception rates and others cannot be attributed exclusively to the type of semen used for insemination. Another recent study on Holstein heifers showed an even better conception rate when using sexed semen (47.3%), despite still slightly inferior to when using unsexed semen (56.9%) (108).

Given that several factors may influence the performance of sexed semen, ensuring profitability entails careful consideration of strategies for successful implementation with favorable financial returns. Since the application of sexed semen is of interest across various species, studies that explored methods to enhance the implementation of sexed semen in cattle farming were reviewed. These studies, whether based on field tests or predictive models, are discussed below, and some major findings are synthesized in Table 3.

**Table 3:** Strategies for cost-effective implementation of sexed semen in the cattle industry.

Aim	Breed	Evidence	Estrus detection	Strategy	Reference
To achieve higher pregnancy rates based on the animal’s reproductive performance	Holstein (heifers and cows)	Experiment	N/A	To prioritize the insemination of heifers instead of cows	(97)
To increase the overall net return and genetic level through a phased implementation of sexed semen	Not specified	Model-based^a^	N/A	To have a herd with high management for reproductive performance and to inseminate 75% of the genetically superior heifers with sexed semen and 70% of the genetically inferior multiparous cows with beef semen. Changes in the genetic level have to be considered	(99)
To achieve the highest total economic return through dairy crossbreeding and genomic test	Swedish Holstein	Model-based^b^	N/A	Use 90% sexed semen in heifers and 45% sexed semen in first-parity cows combined with genomic test and crossbreeding (+58€, 33% crossbreds in the herd)^*^	(109)
	Swedish Red		N/A	Use 90% sexed semen in heifers combined with genomic test and crossbreeding (+94€, 46% crossbreds in the herd)^*^	
To increase the pregnancy rate based on the time of AI	Holstein (dairy heifers)	Experiment	An accelerometer system and a neck collar comprising an electronic identification tag	To inseminate 20.1-24 h after estrus detection, coupled with a temperature-humidity index at the time of artificial insemination below 65	(110)
	Jersey (virgin heifers)		Tail-head chalk (checked twice a day for removal)	To inseminate 16.1–24 h after estrus detection (instead of 12–16 h)	(111)
	Jersey (lactating cows)		Tail-head chalk (checked daily for removal of tail paint or standing estrus) and secondary signs evaluation (increased nervousness and activity, walking the fence line, and swelling and reddening of the vulva)	To inseminate 23–41 h after estrus detection (instead of ≤3 h, 4–12 h, 13–22 h, and ≥42 h)	(112)
	Jersey (dairy heifers)		N/A	To inseminate 60 h after the removal of the intravaginal progesterone device (instead of 54 h)	(113)
	Nelore cows (suckled, multiparous)		N/A	To inseminate 60 h after the removal of the intravaginal progesterone device (instead of 48 h or 36 h) OR To inseminate 0–12 h before ovulation (compared to 12–24 h and >24 h)	

^*^Compared with a pure-breeding scenario, without sexed semen and genomic test; AI, Artificial insemination; N/A, Not applicable.

a Combination of two stochastic simulation models: SimHerd and ADAM.

b Combination of two stochastic simulation models: SimHerd Crossbred (operational returns) and ADAM (genetic returns).

Researchers have investigated how the reproductive performance and proportion of females of the overall herd inseminated with sexed semen can determine the extent of financial returns. As reported by Norman and collaborators (2010), the conception rate and performance of sexed semen may differ between heifers and cows (97). Although the number of inseminations per group varied, as well as other factors since this was a retrospective study, when using sexed semen the mean conception rate for heifers and cows was 39 and 25%, respectively (97). Moreover, Kawano and collaborators (2014) recommended that producers should use sexed semen on at least half of the inseminations and must carefully choose the females to ensure a conception rate of at least 45% [(114) as cited by (102)].

Therefore, the phased implementation of sexed semen, combined with other breeding tools, can enhance the net income and genetic level of the herd. Sexed semen can be used first to inseminate heifers of genetic interest, while conventional semen is used for the others (99, 107, 115). Then, over time, sexed semen can be introduced to manage the overproduction of animals of unwanted sex. Simulation models have been used to predict economic returns in several scenarios. Based on the combination of two stochastic simulation models that take into consideration operational (SimHerd) and genetic (ADAM) returns, net returns can increase if using sexed semen in 75% genetically superior heifers and beef semen in 70% genetically inferior cows (99). However, those gains showed to be herd-specific and only attainable in herds with high management for reproductive performance. In herds with average management levels, net returns decreased. Furthermore, none of the scenarios were viable when the value of the increased genetic level was not taken into account (99). In a more recent study, the possible outcomes in terms of the total economic return in dairy farms were studied. This study focused on the interaction of sexed semen with three additional tools: terminal crossbreeding, genomic testing, and semen of beef breeds (109). The authors concluded that the highest economic return was achieved by combining both genomic testing and crossbreeding, with 90% sexed semen used in heifers and 45% in first-parity cows for Swedish Holstein, or with 90% sexed semen used in heifers for Swedish Red. Moreover, although compensated by the operational return, terminal crossbreeding resulted in lower genetic returns compared to pure-breeding scenarios (109).

Regarding technical factors, some studies have been performed to investigate which would be the optimal timing of insemination to achieve the best pregnancy rates when using sexed semen. Guner et al. inseminated Holstein dairy heifers 12–16 h, 16.1–20 h, or 20.1–24 h after estrus (110). As expected, heifers showed a 12% higher pregnancy rate when AI was performed closer to the ovulation time (20.1–24 h) (110). Previous research has shown results that also support that delaying AI to some extent when using sexed semen promotes higher pregnancy rates, mentioning several time points such as 16.1–24 h (virgin Jersey heifers) and 23–41 h (lactating Jersey cows) after oestrus, and 0–12 h before ovulation (Nelore cows) (111–113). On the other hand, the number of pregnancies per AI did not improve when Chebel et al. delayed the time of insemination with sexed semen by 12 h (71). The authors hypothesized that their results may have been affected by the broad differences between the initiation of estrus and AI among treatments (71).

It should be noted that estrus detection is a critical and not simple step influenced by inter-animal variation. Correct estrus identification may be challenging since it takes a high level of competence to identify estrus signals. Otherwise, this can be reflected in erroneous reports and, consequently, in establishing the wrong optimal period to perform AI. Alternative approaches for estrus detection are available, such as recording body temperature and vaginal mucus resistance changes [reviewed in (116)]. The number of steps also increases closer to estrus. Hence, pedometers can help in properly detecting the onset of estrus and predicting the time of ovulation, contributing to more successful inseminations (117).

On the other hand, the need to monitor and breed each cow individually results in a less streamlined and perhaps less efficient AI program, depending on available resources. Hence, other options, such as hormonal synchronization protocols coupled with a fixed-time AI, are also commonly used and well-documented in both dairy and beef cattle. By synchronizing the estrus cycles of a group of cows, all cows can be inseminated at the same time, allowing better management of insemination timing. It is even described that the total pregnancy rate per breeding season can be higher when synchronizing cows compared to non-synchronized systems (87.5% vs. 75%, respectively), due to the possibility of introducing an extra estrus cycle in a standard 42–44 days breeding season (118). According to a recent study that involved almost 900 heifers/cows from six different herds, synchronized with the 7 d CO-Synch plus CIDR protocol, the combination of Sexcel^™^ gender-ablated semen coupled to a fixed time AI can be used successfully if animals exhibit estrus (60). Diniz and collaborators (2021) compared pregnancy rates in Nelore cows inseminated with sexed semen or conventional semen, with the absence or expression of estrus while using PGF2α at the moment of AI in a progesterone(P4)/estradiol(E2)-based timed AI protocol. They found that cows that evidenced estrus at timed AI showed better fertility and that the use of PGF2α at the time of AI seemed to increase the pregnancy rate in animals that did not exhibit evident signs of estrus, regardless of the semen type. Nevertheless, conventional semen performed better than sexed semen (74). Another study showed that larger POFs were associated with earlier ovulation and demonstrated that the fixed-time AI protocols can therefore be improved by adjusting the time of AI according to the diameter of the POF (75). Several studies are looking at the best protocol for fixed-time AI coupled to sexed semen that can easily be performed on farms, mainly because it does not depend on the accuracy of estrus detection, and treatments available have been previously reviewed (119–121).

The technicians’ experience was also found to influence the success of AI when employing sexed sperm and, therefore, they must be trained regularly (72, 110). Additionally, Oikawa and collaborators (2019) found that during the warmer months (June, July, and August) heifers inseminated with sexed semen had an especially lower conception rate than those inseminated with unsexed semen (108). Therefore, heat stress can be another point to consider for a more careful implementation of AI with sexed semen.

Additionally, some authors suggest the use of sexed semen in combination with *in vitro* embryo production, where oocytes are harvested from a donor female, fertilized *in vitro*, and then the embryos are transferred to recipients (122). The main advantages are that the producer can choose the female and male coupling based on their genetic traits and that fewer sexed spermatozoa are needed per oocyte for *in vitro* fertilization compared to regular AI. This allows for an increase in the number of offspring per female and contributes to accelerated genetic progression while redirecting production solely toward the desired sex (122, 123). Yet, its success also depends on several factors [reviewed in (122)]. The use of this methodology with transfer to beef recipients to obtain sex-specific offspring in cattle has already been described as a viable large-scale production system (122). In a 2016 study, it was observed that pregnancy rates were even better when using sexed semen in combination with fixed-time embryo transfer of *in vitro*-produced embryos in cattle than with fixed-time artificial insemination (124).

We will now discuss the existing literature on the economic benefits of having such technology for other species, beyond cattle, as well as some preliminary results of its use, when available.

#### 4.2.2 Small ruminants

Sheep and goats continue to be among the most consumed species and meat production goes hand in hand with other management systems applicable to these species, including milk and wool production. The prospects are that their market will gradually expand in the upcoming years (51).

In sheep farms where artificial insemination is already a standard practice, in addition to the potential for streamlining flock management and increasing revenue through the sale of rams, adopting a cost-effective insemination protocol with sexed semen could result in reduced expenses related to progeny testing and enhanced genetic progress rates (125, 126). Moreover, woolgrowers would be able to concentrate their selection efforts on a specific ewe lineage (125).

The first attempts to inseminate ewes with sexed semen were not successful or profitable due to challenges related to impaired sperm transport, as well as the high selectivity of the ewe’s cervix [reviewed in (11)]. This was overcome with the development of new insemination technologies, such as laparoscopy where the inside of the abdomen is accessed (11). De Graaf and collaborators investigated the use of laparoscopy combined with sexed semen in Merino ewes that were synchronized with progestagen pessaries, PMSG, and GnRH treatments (11). The ewes were inseminated with varying doses of motile frozen spermatozoa, either sexed (S) or unsexed (U). The doses included 1 × 10^6^ (S1/U1), 5 × 10^6^ (S5/U5), and 15 × 10^6^ (S15/U15) spermatozoa. A control group was included, where ewes were inseminated with 50 × 10^6^ motile unsexed frozen spermatozoa (U50). Ewes’ lambing rate was similar for the U50 (63.2%), U15 (68.5%), S15 (66.7%), S5 (66.1%), and S1 (61.5%) groups. However, the success rate was lower for the U5 (39.5%) and U1 (34.5%) groups. Additionally, the lower doses of sexed semen (S1 and S5) resulted in greater pregnancy rates than the respective unsexed doses (U1 and U5) (11). These findings, according to the authors, have also significant implications for the sexed sperm production industry in sheep. Part of these positive results may be attributed to the fact that the sexed samples were previously analyzed, and only motile spermatozoa were utilized. Additionally, laparoscopic insemination has proved to be of great value in consistently achieving satisfactory results when using sexed semen, especially with low insemination doses (11). However, the equipment is expensive and it requires surgical expertise to be performed [reviewed in (10)]. By reducing the effective number of spermatozoa, the cost of production per dose can be decreased.

The same author also conducted an interesting investigation into the fertilizing capacity of frozen sexed ram semen in superovulated ewes, as well as the quality and survivability of the collected embryos that were transferred immediately into the uterine lumen of synchronized recipients (77). The study involved inseminating ewes with motile sexed (15 × 10^6^ spermatozoa) and unsexed semen (15 × 10^6^ and 30 × 10^6^ spermatozoa) to evaluate the efficacy of the process. Donor animals were synchronized using an intravaginal progestagen pessary and GnRH protocol, and then superovulated with PMSG and FSH before insemination. The study revealed that 92.5% of the embryos were born of the predicted sex after AI with sexed semen. Furthermore, the number of transferable embryos, pregnancy rate, and embryo survival rate were very similar between the two groups of animals. This indicates that the flow cytometry procedure used in sexing the semen did not affect the *in vivo* fertilization capacity of the ram spermatozoa in superovulated ewes, nor did it negatively impact the survivability of the resulting embryos (77). Thus, the results obtained in these de Graaf studies may be useful for the sheep industry’s sustainability and profitability (11).

As far as goats are concerned, in addition to the direct value for dairy farms, this technology also has the potential to expedite herd expansion and enhance its profitability, while maintaining a high level of biosecurity (51, 87). In 2013, it was demonstrated for the first time the successful sorting and cryopreservation of caprine semen, along with the birth of kids through laparoscopy (127). In this specific study, the use of freeze-thawed sexed semen resulted in lower fertility rates when compared to unsexed semen (38% vs. 50%, respectively). Yet, other studies have reported similar conception rates to those observed with unsexed semen, ranging from 36 to 57%. These rates depended on the specific study conditions, including the number of spermatozoa per dose and whether the semen was fresh or frozen [reviewed in (51)]. Nevertheless, it should be mentioned that laparoscopy is restricted in certain countries due to concerns about potential increases in animal stress and, ultimately, pain [as cited by (87)].

Transcervical insemination is also considered, as goats are the most suitable among small ruminants for this procedure, although the level of difficulty may vary between breeds. However, it is not yet well defined whether this method yields similar pregnancy rates as the traditional higher doses of unsexed semen [reviewed in (51)].

The combination of AI with embryo transfer programs and estrus detection has also been a great ally for goat dairy farms, allowing for genetic improvement and reducing generation interval. Similar to what has been described for other species, integrating these programs with sexed semen may also contribute to the profitability of production (128).

Studies conducted in the context of improving the implementation of sexed semen for small ruminants were recently reviewed by González-Marín et al. (51). The authors emphasize that while the small ruminant industries have begun testing the use of sexed semen in recent years, further research is required to determine the optimal synchronization protocols, timing of AI, and the ideal insemination dose (51).

#### 4.2.3 Pigs and rabbits

Besides the advantages already discussed for swine and rabbit production, the utilization of sexed semen can also be beneficial for both species due to differences in the palatability of meat between females and males (5, 88, 90). Particularly in swine production, to prevent boar taint and ensure the consumption of male meat, producers routinely perform castration. However, because regular castration without anesthesia or analgesia is likely to be outlawed in the future and the sensory perception of eating quality is important, immunocastration and new ways of detecting boar taint, such as the detection at-line with laser diode thermal desorption tandem mass spectrometry, have arisen as a more animal-friendly option (88). Still, the need to adjust slaughter lines and the considerable investment required to implement this technique make the investment in female production a desirable economic and welfare option (88, 90). According to a recent study on crossbred pigs, if slaughtered with 105 Kg or 117 Kg, females (gilts) allow for one of the highest gross margins per pig operation per year (GMppy; 74€ and 72€, respectively) compared to castrated males (barrows), entire males, and immunocastrated males. Only entire males of 117 Kg allow a higher GMppy (80€). The fact that weight contributes to production efficiency should not be overlooked, as slaughtering gilts that weigh 130 Kg can represent a 60% decrease in the GMppy (129). Also, Serrano and coworkers (2008) reported that intact female pigs were more profitable for intensive production of Iberian pigs than castrated males and castrated females. These results are supported by the fact that they eat less and have less carcass fat and more primal cuts yield. They also tend to have greater feed conversion (130).

Nonetheless, most female pigs are artificially inseminated with refrigerated semen (~1–3 billion spermatozoa/dose, depending on the AI procedure), since boar cryopreserved semen has limited efficiency. Therefore, the implementation of sex-sorted semen still presents some challenges, not only because sex-sorted semen is commonly cryopreserved, but also because a particularly high number of spermatozoa per insemination dose is required for AI in pigs [reviewed in (37, 131)]. Laparoscopic insemination, by allowing sperm deposition directly into the utero-tubal junction and/or the oviduct, has already been described as an option for successful inseminations using only 1–3 million liquid-stored sexed spermatozoa. Despite fertility rates of over 70% and convenient insemination doses, this method is very costly, requires skills, and needs to be applied shortly before ovulation for maximum fertility [reviewed in (10, 131)]. Thus, this may represent an opportunity for sperm sexing kits that can be applied to both fresh and cryopreserved semen, like the WholeMom kit for sows and the EMLAB Genetics’ PIGPLUS^™^ and BOARPLUS^™^ kits, mentioned previously, even though there is not much available data regarding their performance and success.

In the case of rabbits, male meat also tends to have a stronger odor than female meat, possibly explained by significantly different concentrations of volatile compounds and, therefore, certain individuals may prefer female rabbit meat (132, 133). In terms of meat quality, studies tend to show similar results when comparing meat from both sexes, but a recent study showed higher vitamin A content in female meat [reviewed in (134)]. The aroma flavor characteristics of cooked meat play one of the most important roles in acceptance and preference by consumers, and therefore, since the market position is accompanied by consumers’ preference (135), aroma desirability may bring a possible problem for rabbit production itself, if a preference for female meat prevails. This can also undermine the financial return that companies focused on selling does for breeding purposes could get from surplus males.

#### 4.2.4 Horses

For instance, in horses, the artificial insemination procedure alone is more labor-intensive, with conventional *in vitro* fertilization yielding limited success, primarily due to the sperm’s inability to penetrate the oocyte, often because of incomplete capacitation (76). Nevertheless, in addition to the available immunological kits, it is possible to find few equine breeding centers around the world offering sperm sexing options, such as sexed fresh semen for mares residing on specific farms or sexed frozen semen for use in Intracytoplasmic Sperm Injection (ICSI) programs, with pricing packages starting at approximately 1,000€ (136).

ICSI offers the advantage of requiring only one selected spermatozoon that is injected directly into the oocyte, enabling the use of semen samples even if they contain a lower number of viable sperm. This may be particularly relevant with stallion frozen–thawed semen and sexed semen samples. Nonetheless, this type of selection bypasses natural processes, allowing for the possibility of choosing sperm with DNA damage or abnormal internal structure, which could potentially have adverse effects on embryo development [reviewed in (76)].

According to some other studies performed in mares, when inseminating them into the tip of the uterine horn ipsilateral to the preovulatory follicle with 25 million sexed or unsexed progressive motile spermatozoa pregnancy, rates of 30–50, and 57% were obtained, respectively (137). In another study that used the same method of insemination coupled with an insemination dose of 40 million sexed spermatozoa (45–55% progressively motile), a pregnancy rate of 54% was obtained (138). In other studies, while employing hysteroscopic insemination, a similar pregnancy rate of around 40% was observed when using either 5 million sexed or unsexed motile spermatozoa (139), and a pregnancy rate of around 72% was achieved when using 20 million refrigerated (15°C) sexed spermatozoa (140).

Therefore, based on these limitations and preliminary results, sperm sexing is still not widely applied in horses (12, 13).

As we delve into the implementation of sexing technologies, independently of the species, we should be aware that it may represent an increase in the final cost for consumers. However, the implications on the final cost of products derived from welfare-friendly productions are not universally accepted, although the welfare status of animals is important for consumers. As shown in the 2015 European Commission survey, around 59% of participants are willing to pay more for these types of products. Nevertheless, 35% of them are only happy to pay up to 5% more than current prices, while a sizable 35% of European citizens do not want to have additional expenses (79). Therefore, to address the challenges associated with rising prices, farmers must have a close relationship with researchers so that technologies can be improved to the point where their application does not affect sales.

### 4.3 Environment

Climate change, endangered species, and livestock production must go hand in hand since not only animal production can impact the environment but also animals can be affected by climate changes.

Therefore, climate change mitigation strategies must go through adaptation in animal production (33, 141). Part of the negative impact of livestock on the environment is related to animal feeding and the emission of GHG (142). Ruminant CH_4_ emissions and the impact of those enteric gases, for example, are a subject of public discussion (141). Those emissions and their increase are related to the amount of forage digested and high levels of performance (141, 142). Although different types of production imply different levels of GHG, some mitigation measures were already mentioned in animal production, such as the reduction of livestock, efficient use of animal resources, and optimization of feed rationing (141). Inevitably, promoting the development of feed of better quality with optimized feed additives would help in this matter (3, 141). Also, genetic selection methods represent an opportunity to reduce GHG emissions, but other options are available (141). If we could enhance reproduction and replacement rates, as well as the performance of each animal, we would need fewer animals per unit of output products (142). In the case of productions directed to one of the sexes, sexed semen would be an appropriate tool since it would contribute to the depletion of the less productive individuals of the herd and their inherent environmental impact (143). Therefore, improving production efficiency through reproduction can be one of the keys to reducing the environmental impact.

Moreover, sexed semen may play a role in the conservation and sustainability of endangered species by helping increase their populations and control gender balance, which is essential for biodiversity preservation and the long-term health of ecosystems. As a case in point, it is possible to consider the rare horse breed with less than 300 females globally, the Suffolk Punch. This underscores the critical significance of expediting the augmentation of female numbers. In a collaborative project between the conservation charity “The Rare Breed Survival Trust” and Nottingham Trent University in 2020, the first filly foal was born after inseminating a Suffolk Punch mare with sexed X-sperm, and the team hopes it will serve as a model for future projects (144).

## 5 Future perspectives and conclusions

It is paramount to promote a sustainable animal industry and responsible consumption while assuring an adequate supply of animal-based protein for balanced nutrition. While we cannot eradicate all animal welfare and environmental issues, we can contribute to minimizing them.

The development of affordable and non-invasive sperm sexing technologies that have minimal impact on sperm quality will be of utmost importance. This would enable a cost-effective application of the technology to a wide range of species for which the pre-selection of offspring sex would benefit the production. Investing in the training and professional development of technicians, delaying AI when using sexed semen compared to when using conventional semen, and combining AI with sexed or conventional semen based on the physical and genetic traits of the females are strategies that can ensure a more successful implementation of sexed semen. Model-based studies can also be an excellent tool for predicting which conditions are required for a profitable employment of sexed semen.

Equally important is to set up collaborations between companies and academics. Communication is essential. The combination of the know-how of both worlds will enable the refinement of such technologies and the establishment of guidelines for a worthwhile implementation in different systems, revolutionizing breeding management. Partnerships should also go beyond that. Academics must guarantee that their scientific findings are disseminated not only among the scientific community but also to the business community, which may benefit from those scientific breakthroughs. On the other hand, producers should increasingly seek information from the scientific community, veterinarians, commercial partners, and other farmers to improve the way they operate.

Concurrently, a more widespread of sexed semen in farms would promote the incorporation of reduction and refinement principles in several animal production sectors, including beef and dairy cattle, ovine, caprine, swine, and rabbit productions, translating into more efficient, competitive, profitable, and animal-friendly industries.

## Author contributions

JQ, PP-P, and MF: conceptualization. PP-P: methodology. JQ and PP-P: investigation and writing – original draft. MF, GL, AR, RP-L, and BC: writing – review & editing. MF and BC: supervision. All authors contributed to the article and approved the submitted version.
